# Incidence of Gastrointestinal Bleeding After Transesophageal Echocardiography Use in Orthotopic Liver Transplantation

**DOI:** 10.3389/ti.2022.10753

**Published:** 2022-10-20

**Authors:** Minesh Chotalia, Upasana Topiwala, Asim Iqbal, Dhruv Parekh, John L. Isaac, M. Thamara P. R. Perera, Mohammed A. Arshad

**Affiliations:** ^1^ Department of Anaesthetics and Critical Care, Queen Elizabeth Hospital Birmingham, Birmingham, United Kingdom; ^2^ Birmingham Acute Care Research Group, University of Birmingham, Birmingham, United Kingdom; ^3^ Department of Anesthesiology and Perioperative Care, Vancouver General Hospital, University of British Columbia, Vancouver, BC, Canada; ^4^ Department of Liver Surgery, University Hospitals Birmingham NHS Foundation Trust, Birmingham, United Kingdom; ^5^ Department of Liver Surgery, Birmingham Children’s Hospital NHS Foundation Trust, Birmingham, United Kingdom

**Keywords:** liver transplantation, cardiovascular, liver, transesophageal echocardiography, echocardiography

## Abstract

The risk of upper gastrointestinal bleeding (UGIB) after transesophageal echocardiography (TEE) in patients with high grade esophageal varices (EV) that are undergoing Orthotopic Liver transplantation (OLT) is poorly understood. This was a retrospective single-centre cohort study in all patients that underwent OLT at Queen Elizabeth Hospital Birmingham between September 2016 and September 2018. The primary outcome was to determine the incidence of UGIB in patients that have undergone OLT with EV that received TEE. 401 patients were included in the study, of which 320 (80%) received TEE. The incidence of post-operative UGIB in patients that received TEE was 1.6% (5/320) in the entire cohort: 2.7% (4/149) in patients with no evidence of EV and 0.6% (1/171) in patients with EV. UGIB occurred in 1 patient with grade 2 EV and did not occur in patients with grade 1 or 3 EV. The incidence of UGIB in patients that received TEE was not statistically different to patients that did not: 1.6% (5/320) vs. 3.7% (3/81) p = 0.218. In conclusion, in patients that underwent OLT, intra-operative TEE use was associated with low rates of UGIB, even in cohorts with high grade EV. This suggests that TEE is a relatively safe method of haemodynamic monitoring in patients undergoing OLT.

## Graphical Abstract

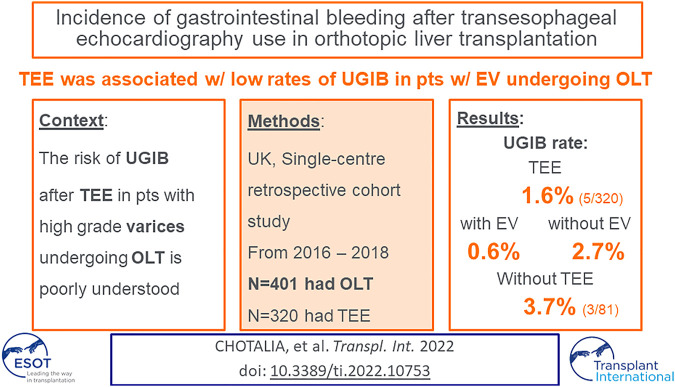

## Introduction

Cardiovascular instability is common during Orthotopic Liver Transplantation (OLT) and may be precipitated by cross-clamping the inferior vena cava and portal vein, surgical manipulation and reperfusion [1]. Haemodynamic monitoring is therefore vital in administering fluid/blood products and vasoactive agents during OLT and transesophageal echocardiography (TEE) is increasingly being utilised in this regard [1]. TEE has the ability to quickly detect rare but devastating intraoperative complications during OLT, such as intracardiac thrombosis and pulmonary embolism, as well as guide therapy for them. Numerous studies have demonstrated the effectiveness of TEE in the diagnosis and treatment of cardiovascular perturbations during OLT [2].

Large multicentre studies have established that TEE is a relatively safe procedure [3,4], but patients with esophageal varices (EV) were excluded from these analyses as TEE has previously been considered relatively contraindicated in this patient cohort due to concerns of precipitating upper gastrointestinal bleeding (UGIB) [5]. Recently small retrospective studies in patients with EV that received TEE demonstrated similarly low rates of UGIB, however the number of patients with high grade EV (grade 2–3) were small [6,7,8,9,10,11]. As the risk of bleeding is proportional to the size of the varix [12], this is an important omission.

As EV are present in almost 3/4 patients with end-stage liver disease (ESLD) awaiting OLT [13] and bleeding from EV is a serious complication with a 20% mortality rate [14], evaluating the safety of TEE in patients with high grade varices undergoing OLT is of paramount importance. Therefore, the main aim of this study was to determine the incidence of UGIB in patients with EV that received TEE during OLT. Secondary aims were to compare the rates of UGIB in patients with different grades of varices and in patients that underwent OLT with and without TEE.

## Methods

### Ethical Approval

This study was a retrospective service evaluation of anonymised, routinely collected data as defined by the UK NHS Health Research Authority (http://www.hra.nhs.uk). The study was registered with the hospital’s clinical audit registration system (CARMS-14529) and specific ethical permissions were not required.

### Data Collection

This was single-centre retrospective cohort study of patients that underwent OLT at the Queen Elizabeth Hospital Birmingham (University Hospitals Birmingham NHS Foundation Trust) between September 2016 and September 2018. Data were retrieved retrospectively from the hospital’s electronic patient records, surgical and anaesthetic records and included demographic data, MELD score, blood test results on the day of OLT (biochemistry, full blood count and coagulation profile), blood product transfusion during OLT and medical history of previous EV treatments including beta blocker, transjugular intra-hepatic portosystemic shunt (TIPSS) insertion, band ligation or sclerotherapy. Varices were graded in accordance with the modified Paquet classification [15]. UGIB was defined as the presence of blood in the oesophagus or stomach at the time of oesophago-gastric duodenoscopy. Clinically significant UGIB was defined by a transfusion requirement of packed red cells or if there was a drop in haemoglobin of >2 g/dl.

### Statistical Analysis

All statistical analysis was performed using GraphPad Prism v.8.0. Categorical data are presented as n (%) and compared using a chi squared test. Continuous data were tested for normality using Shapiro-Wilk’s test. If not normally distributed, continuous were presented as median (interquartile range) and were compared using a Mann-Whitney U test. This was a pragmatic study and post-hoc power calculations to determine study size were not performed. All tests performed were two-sided and a *p* value < 0.05 was considered statistically significant.

### Transesophageal Echocardiography

The decision to perform a TEE was at the discretion of the treating consultant liver transplant anaesthetist. The echocardiogram was conducted and interpreted by this anaesthetist, who had relevant experience in perioperative TEE use. A standardised protocol of obtaining mid-esophageal and transgastric views was followed using a Phillips TEE probe and Phillips CX50 ultrasound machine (Phillips Healthcare, Andover, MA, United States). The TEE probes were routinely inserted after induction of anaesthesia and placement of an endotracheal tube and withdrawn at the closure of the abdomen at the end of the surgery.

## Results

401 patients were included in the study and had a median age of 56 (IQR 46–64), were 66% male and had a median MELD score of 14 (IQR 10–19). The most common indication for OLT was alcoholic cirrhosis (*n* = 119, 30%) and the most common graft type was donation after brain death (DBD) (*n* = 267; 67%). ICU mortality for the entire cohort was 4% (*n* = 15). Additional demographics are listed in Table 1.

**TABLE 1:** Comparing clinical and demographic parameters in OLT patients that did and did not receive TEE.

Demographic	All (*n* = 401)	Received TEE (*n* = 320)	No TEE (*n* = 81)	*p* value
Age (years)	56 (46–64)	56 (46–64)	57 (46–64)	0.975
Sex (%male)	263 (65.6)	210 (65.6)	53 (65.4)	0.755
MELD score	13.9 (10.2–18.8)	14.1 (10.2–18.9)	13.8 (10.2–15.8)	0.283
Indication for OLT				0.757
Alcoholic	104 (25.9)	80 (25.0)	24 (29.6)	
PSC	68 (17.0)	53 (16.6)	15 (18.5)	
NASH	54 (13.5)	45 (14.1)	9 (11.1)	
PBC	41 (10.2)	35 (10.9)	6 (7.4)	
Hepatitis C	26 (6.5)	21 (6.6)	5 (6.2)	
Other	108 (26.9)	86 (26.9)	22 (27.2)	
Grade of varices				0.228
None	193 (48.1)	149 (46.6)	44 (54.3)	
1	133 (33.2)	107 (33.4)	26 (32.1)	
2	61 (15.2)	54 (16.9)	7 (8.6)	
3	14 (3.5)	10 (3.1)	4 (4.9)	
Bilirubin μmol/L	35 (17–64)	35 (16–65)	37 (17–57)	0.905
INR	1.4 (1.2–1.6)	1.4 (1.2–1.6)	1.3 (1.2–1.5)	0.330
Platelets x10^9^/L	92 (65–144)	91 (65–143)	105 (66–154)	0.435
Donor type (%DBD)	267 (66.6)	212	55	0.778
Blood product transfusion (units)				
Packed red cells	2 (0–4)	2 (0–4)	2 (0–4)	0.674
FFP	4 (0–6)	4 (0–6)	4 (0–6)	0.771
Platelets	1 (0–10)	1 (0–5)	0 (0–5)	0.145
Cryoprecipitate	0 (0–0)	0 (0–0)	0 (0–0)	0.210
Cell saver (mls)	450 (0–780)	450 (0–770)	460 (0–990)	0.924
UGIB incidence	8 (2.0)	5 (1.6)	3 (3.7)	0.218
OGD performed	18 (4.5)	14 (4.4)	4 (4.9)	0.827
ICU mortality	15 (3.7)	10 (3.1)	5 (6.1)	0.197

Legend: OLT, orthoptic liver transplantation; TEE, transesophageal echocardiography; PSC, primary sclerosing cholangitis; PBC, primary biliary cirrhosis; NASH, non-alcoholic steatohepatitis; MELD, model for end-stage liver disease; INR, international normalized ratio; DBD, death brain stem donation; FFP, fresh frozen plasma; UGIB, upper gastro-intestinal bleeding; OGD, oesophago-gastric duodenoscopy; ICU, intensive care unit.

Of the 401 patients, 320 (80%) received TEE. Of these patients, 149 (47%) had no evidence of EV, 107 (33%) had grade 1 EV, 54 (17%) had grade 2 EV and 10 (3%) had grade 3 EV. No episodes of intra-operative UGIB occurred. The incidence of post-operative UGIB in patients that received TEE was 1.6% (5/320) in the entire cohort: 2.7% (4/149) in patients with no evidence of EV and 0.6% (1/171) in patients with EV. A post-operative UGIB occurred in 1 patient with grade 2 EV, however this was not associated with a drop in haemoglobin or red blood cell transfusion. An UGIB did not occur in patients with grade 1 or 3 EV. The rates of UGIB were not statistically different between patients with and without EV and across different grades of EV. There were no incidences of clinically significant UGIB in patients that underwent TEE. Patients with high MELD scores (≥18) had no statistically significant difference in UGIB incidence compared to those with low MELD scores (<18; 2/93 (2.2%) vs 3/227 (1.3%); *p* = 0.630).

### Comparison to Patients That did not Receive TEE

81 patients underwent OLT but did not receive a TEE. There were no differences in the demographics or incidence of EV between patients that did and did not receive TEE (Table 1). The incidence of UGIB in patients that received TEE was not statistically different to patients that did not [1.6% (5/320) vs 3.7% (3/81); *p* = 0.218]. The number of blood products transfused intra-operatively were also similar between cohorts, as was the ICU-mortality rate.

## Discussion

In one of the largest studies in this field to date, we demonstrate a low rate of gastro-intestinal bleeding (<1%) following TEE in patients with EV undergoing liver transplantation. This relatively low risk of bleeding was also present in patients with high grade EV (Grade 2 or 3; 1.6%), a cohort that has previously been sparsely assessed in the literature. Furthermore, the rate of UGIB in patients that received TEE was no different to those that did not receive TEE during their OLT. Altogether, this suggests the relative safety of this semi-invasive monitoring technique in patients undergoing OLT, although larger, multi-centre studies are required to validate these findings. It is worth noting that this patient cohort (by definition) are all intubated, have excellent IV access and have available cross matched blood prior to TEE insertion. This provides a safety net should UGIB occur.

## Comparison to Previous Literature

Numerous large, multi-centre studies have demonstrated the relative safety of TEE, with GI bleeding rates of 0.02–1% and a GI tract perforation risk of 0.01% [3, 4]. However, these studies largely excluded patients with EV, likely secondary to the historic recommendation that the presence of portal hypertension or EV were relative contraindications to TEE examination [5]. Since then, smaller retrospective studies have demonstrated a low bleeding risk following TEE in hospitalised patients with EV [6–9]. However, to the best of our knowledge, only one patient with grade 3 varices was included in these studies. Furthermore, portal venous pressures during the process of liver transplantation are likely to be markedly different to hospitalised patients with EV, hence the risk profile identified in these studies may not be directly applicable to TEE use in OLT.

In patients undergoing OLT with varices, a similarly low risk of GI bleeding following TEE was identified by Burger-Klepp et al [10] and Pai et al [11], however only 7 patients had Grade 3 varices. Here we identified 171 patients with EV, 10 of whom had grade 3 varices and also demonstrated a <1% risk of GI bleeding with TEE in these patients. If data from all of these studies are combined, the rate of UGIB following TEE is 0.2% (3/619) in patients with EV undergoing OLT. In the present study, there were no incidences of clinically significant UGIB (necessitating > 2 units packed red blood cell transfusion or drop in haemoglobin by 2 g/dl) following TEE. Importantly, this is also the first study to our knowledge to demonstrate equivalent UGIB rates in patients that underwent OLT with and without TEE, suggesting that the rates of bleeding identified may be independent of TEE use. This finding is corroborated by reports that variceal rupture is precipitated more commonly by intrinsic pressure in the portal system, after clamping the portal system at the start of the anhepatic phase, rather than direct external pressure [15]. Unfortunately, we were unable to analyse the duration of the anhepatic phase comprehensively in all patients to test this hypothesis. Furthermore, rates of UGIB were equivalent in patients with and without EV, suggesting that the presence of EV should not be a contraindication to intra-operative TEE examination during liver transplantation.

### Strengths and Limitations

Despite assessing bleeding risk following TEE in the largest number of patients with grade 3 EV to date, the small patient numbers with high grade EV and low event rate of UGIB means that the study lacks sufficient power to detect clinically significant complications of TEE in this patient cohort and is at risk of type 2 statistical error. The retrospective nature of the study may have led to reporting bias, with only clinically significant bleeding being documented in the notes. Nevertheless, occult UGIB that does not precipitate OGD examination, RBC transfusion or drop in haemoglobin, is unlikely to contribute significantly to patient morbidity. The cohort had lower median MELD scores (13.9 (IQR 10.2–18.8) than other published OLT cohorts [8–11] and therefore the generalisability of these findings may not extend to patients with very severe hepatic insufficiency. However, there was no statistically significant difference in the incidence of UGIB in patients with high MELD scores (≥18) compared to low MELD scores (<18). We therefore have no evidence to suggest that TEE is unsafe/precipitates UGIB in patients with a greater severity of hepatic insufficiency. Lastly, the study may have been influenced by selection bias, as the choice to perform TEE was at the discretion of the treating anaesthetist, and patients that did not receive TEE may have had a clinically perceived increased risk of variceal bleeding. However, variceal grade, severity of liver disease and markers of coagulopathy did not differ between patients that did and did not receive TEE.

## Conclusion

In patients that underwent OLT, intra-operative TEE use was associated with low rates of UGIB, even in cohorts with high grade EV. This suggests that TEE is a relatively safe method of haemodynamic monitoring in patients undergoing OLT.

## Data Availability

The raw data supporting the conclusion of this article will be made available by the authors, without undue reservation.

## Abbreviations

UGIB, upper gastrointestinal bleeding; TEE, transesophageal echocardiography; EV, esophageal varices; OLT, orthotopic liver transplantation; TIPPS, transjugular intrahepatic portosystemic shunt; DBD, donation after brain death.

## References

[1] Burtenshaw AJ Isaac JL. The Role of Trans‐oesophageal Echocardiography for Perioperative Cardiovascular Monitoring during Orthotopic Liver Transplantation. Liver Transpl (2006) 12(11):1577–83. 10.1002/lt.20929 17058248

[2] Zerillo J Hill B Kim S DeMaria S Jr Mandell MS. Use, Training, and Opinions about Effectiveness of Transesophageal Echocardiography in Adult Liver Transplantation Among Anesthesiologists in the United States. Semin Cardiothorac Vasc Anesth (2018) 22(2):137–45. 10.1177/1089253217750754 29303422

[3] Kallmeyer IJ Collard CD Fox JA Body SC Shernan SK. The Safety of Intraoperative Transesophageal Echocardiography: a Case Series of 7200 Cardiac Surgical Patients. Anesth Analg (2001) 92(5):1126–30. 10.1097/00000539-200105000-00009 11323333

[4] Daniel WG Erbel R Kasper W Visser CA EngbeRding R Sutherland GR Safety of Transesophageal Echocardiography. A Multicenter Survey of 10, 419 Examinations. Circulation (1991) 83(3):817–21. 10.1161/01.cir.83.3.817 1999032

[5] Hahn RT Abraham T Adams MS Bruce CJ Glas KE Lang RM Guidelines for Performing a Comprehensive Transesophageal Echocardiographic Examination: Recommendations from the American Society of Echocardiography and the Society of Cardiovascular Anesthesiologists. Anesth Analg (2013) 26(9):921–68. 10.1213/ANE.0000000000000016 23998692

[6] Spier BJ Larue SJ Teelin TC Leff JA Swize LR Borkan SH Review of Complications in a Series of Patients with Known Gastro-Esophageal Varices Undergoing Transesophageal Echocardiography. J Am Soc Echocardiogr (2009) 22(4):396–400. 10.1016/j.echo.2009.01.002 19231133

[7] Pantham G Waghray N Einstadter D Finkelhor RS Mullen KD. Bleeding Risk in Patients with Esophageal Varices Undergoing Transesophageal Echocardiography. Echocardiography (2013) 30(10):1152–5. 10.1111/echo.12274 23742625

[8] Liu E Guha A Dunleavy M Obarski T. Safety of Transesophageal Echocardiography in Patients with Esophageal Varices. J Am Soc Echocardiogr (2019) 32(5):676–7. 10.1016/j.echo.2019.01.013 30846321

[9] Hudhud D Allaham H Eniezat M Enezate T. Safety of Performing Transoesophageal Echocardiography in Patients with Oesophageal Varices. Heart Asia (2019) 11(2):e011223. 10.1136/heartasia-2019-011223 31275433 PMC6579576

[10] Burger-Klepp U Karatosic R Thum M Schwarzer R Fuhrmann V Hetz H Transesophageal Echocardiography during Orthotopic Liver Transplantation in Patients with Esophagoastric Varices. Transplantation (2012) 94(2):192–6. 10.1097/TP.0b013e31825475c2 22728290

[11] Pai SL Aniskevich S Feinglass NG Ladlie BL Crawford CC Peiris P Complications Related to Intraoperative Transesophageal Echocardiography in Liver Transplantation. Springerplus (2015) 4(1):480–8. 10.1186/s40064-015-1281-3 26361581 PMC4559558

[12] Wadhawan M Dubey S Sharma BC Sarin SK. Hepatic Venous Pressure Gradient in Cirrhosis: Correlation with the Size of Varices, Bleeding, Ascites, and Child's Status. Dig Dis Sci (2006) 51(12):2264–9. 10.1007/s10620-006-9310-2 17080245

[13] Zaman A Hapke R Flora K Rosen H Benner K. Prevalence of Upper and Lower Gastrointestinal Tract Findings in Liver Transplant Candidates Undergoing Screening Endoscopic Evaluation. Am J Gastroenterol (1999) 94(4):895–9. 10.1111/j.1572-0241.1999.984_g.x 10201453

[14] Stokkeland K Brandt L Ekbom A Hultcrantz R. Improved Prognosis for Patients Hospitalized with Esophageal Varices in Sweden 1969–2002. Hepatology (2006) 43(3):500–5. 10.1002/hep.21089 16496319

[15] Paquet KJ. Prophylactic Endoscopic Sclerosing Treatment of the Esophageal wall in Varices-A Prospective Controlled Randomized Trial. Endoscopy (1982) 14(01):4–5. 10.1055/s-2007-1021560 7035153

